# Air pollution in the week prior to delivery and preterm birth in 24 Canadian cities: a time to event analysis

**DOI:** 10.1186/s12940-018-0440-8

**Published:** 2019-01-03

**Authors:** David M. Stieb, Eric Lavigne, Li Chen, Lauren Pinault, Antonio Gasparrini, Michael Tjepkema

**Affiliations:** 1 Environmental Health Science and Research Bureau, Health Canada, 101 Tunney’s Pasture Driveway, Ottawa, ON K1A 0K9 Canada; 2 School of Epidemiology and Public Health, University of Ottawa, Room 101, 600 Peter Morand Crescent, Ottawa, ON K1G 5Z3 Canada; 3 Water and Air Quality Bureau, Health Canada, 269 Laurier Avenue W, Ottawa, ON K1A 0K9, Mail Stop 4903B Canada; 4 Health Analysis Division, Statistics Canada, 100 Tunney’s Pasture Driveway, Ottawa, ON K1A 0T6 Canada; 5 Department of Social and Environmental Health Research, London School of Hygiene & Tropical Medicine, Room 213, 15-17 Tavistock Place, London, WC1H 9SH UK

**Keywords:** Preterm birth, Air pollution, Time-to-event

## Abstract

**Background:**

Numerous studies have examined the association between air pollution and preterm birth (< 37 weeks gestation) but findings have been inconsistent. These associations may be more difficult to detect than associations with other adverse birth outcomes because of the different duration of exposure in preterm vs. term births, and the existence of seasonal cycles in incidence of preterm birth.

**Methods:**

We analyzed data pertaining to 1,001,700 singleton births occurring between 1999 and 2008 in 24 Canadian cities where daily air pollution data were available from government monitoring sites. In the first stage, data were analyzed in each city employing Cox proportional hazards models using gestational age in days as the time scale, obtaining city-specific hazard ratios (HRs) with their 95% confidence intervals (CIs) expressed per interquartile range (IQR) of each air pollutant. Effects were examined using distributed lag functions for lags of 0–6 days prior to delivery, as well as cumulative lags from two to six days. We accounted for the potential nonlinear effect of daily mean ambient temperature using a cubic B-spline with three internal knots. In the second stage, we pooled the estimated city-specific hazard ratios using a random effects model.

**Results:**

Pooled estimates across 24 cities indicated that an IQR increase in ozone (O_3_, 13.3 ppb) 0–3 days prior to delivery was associated with a hazard ratio of 1.036 (95% CI 1.005, 1.067) for preterm birth, adjusting for infant sex, maternal age, marital status and country of birth, neighbourhood socioeconomic status (SES) and visible minority, temperature, year and season of birth, and a natural spline function of day of year. There was some evidence of effect modification by gestational age and season. Associations with carbon monoxide, nitrogen dioxide, particulate matter, and sulphur dioxide were inconsistent.

**Conclusions:**

We observed associations between daily O_3_ in the week before delivery and preterm birth in an analysis of approximately 1 million births in 24 Canadian cities between 1999 and 2008. Our analysis is one of a limited number which have examined these short term associations employing Cox proportional hazards models to account for the different exposure durations of preterm vs. term births.

**Electronic supplementary material:**

The online version of this article (10.1186/s12940-018-0440-8) contains supplementary material, which is available to authorized users.

## Introduction

Preterm birth is a key determinant of infant mortality and morbidity, and of health status in childhood and even adulthood [1–3]. Numerous studies conducted worldwide have examined the association between air pollution and preterm birth [4–10]. Many studies have found that air pollution exposure increases the risk of preterm birth and it has been estimated that 23% or 3.4 million preterm births globally were attributable to PM_2.5_ in 2010 [1]. However, there has been some inconsistency in findings, including in Canada, where in some instances we observed significant associations [11], while in others we did not [12, 13]. Most studies have employed cohort or case-control designs, characterizing exposure over the entire pregnancy, trimester or birth month [8], while a smaller number have examined short term exposure, employing time-series [14–20], case-crossover [21] or time to event analysis [22–26]. It has been hypothesized that the association between air pollution and preterm birth may be more difficult to detect than associations with other outcomes such as term low birth weight or small for gestational age because of the different duration of exposure over the entire pregnancy or third trimester in preterm vs. term births, and the existence of seasonal cycles in incidence of preterm birth [15, 21, 27, 28]. To address these issues and to examine the influence of short-term exposure, here we employ a time to event analysis, using Cox models examining exposures in the week prior to birth.

## Methods

We employed data from the Canadian births database. Live birth events are reported to Statistics Canada by the provincial and territorial Vital Statistics Registries in Canada. For this study, singleton live births between 1999 and 2008 in 24 cities with daily air pollution data were eligible. Data include more than one birth to the same mother, but these could not be identified due to data limitations. Preterm births were those occurring at less than 37 weeks gestation, which were further categorized as 20–27, 28–31, 32–33 and 34–36 weeks gestation [29]. Information on maternal behaviours including smoking and alcohol consumption, and individual-level data on socioeconomic status (SES) and ethno-cultural origins were not available in this dataset. Area-level socioeconomic status characteristics were assigned to singleton births by geocoding birth records using the six character maternal postal code from the births database and the Postal Code Conversion File Plus (PCCF+) version 5 k in order to obtain Statistics Canada standard geographic identifiers [30]. Using geocoded birth records, neighbourhood-level SES variables were calculated at the Dissemination Area (DA) level using census data, including the proportion of individuals aged 15 years and over who were unemployed, or in the lowest income quintile, and the proportion of females aged 25 years and over with post-secondary education [31, 32]. Proportion of individuals in a DA who were classified as visible minority was also calculated. Visible minority groups are defined by the Canadian Employment Equity Act and classification of individuals is based on response to census questions pertaining to self-identified population group [33]. Neighbourhood-level variables were calculated based on the census year closest to the date of birth (2001 or 2006). There were 52,993 and 54,626 DAs in the 2001 and 2006 censuses respectively. Based on the 2006 census, the median and 70th percentile of DA population and land area were 513, 598, 0.26 km^2^ and 1.27 km^2^ respectively.

Daily air pollution data were obtained from the National Air Pollution Surveillance (NAPS) monitoring network for particulate matter of median aerodynamic diameter less than 2.5 μm (PM_2.5_) as well as carbon monoxide (CO), nitrogen dioxide (NO_2_), ozone (O_3_), and sulfur dioxide (SO_2_). Daily temperature data were obtained from Environment and Climate Change Canada’s meteorology data archive. Where data were available from multiple monitors, they were averaged.

Statistical analysis was conducted in two stages. In the first stage, data were analyzed in each city employing Cox proportional hazards models using gestational age in days as the time scale, obtaining city-specific hazard ratios (HRs) with their 95% confidence intervals (CIs) expressed per interquartile range (IQR) of each air pollutant. We tested the proportional hazards assumption using the cox.zph function in R, which evaluates the significance of the interaction between the scaled Schoenfeld residuals for the air pollution term(s) and time, and found no evidence of violation of the proportional hazards assumption. Effects of air pollution were examined using distributed lag functions [34, 35] for lags of 0–6 days prior to delivery, as well as cumulative lags from two to six days. Specification of the lag structure for air pollution and temperature was based on natural spline functions employing three to five degrees of freedom, optimality of which was evaluated based on model Akaike Information Criterion (AIC). We evaluated the optimal lag response specification for O_3_ and temperature in three cities representing diverse climates: Toronto (central Canada), Edmonton (north) and Vancouver (coastal). Three degrees of freedom in the natural spline of both O_3_ and temperature exhibited the lowest AIC for all three cities. We therefore employed this lag structure specification in all 24 cities. Potential non-linearity in associations with air pollution was assessed by specifying air pollution as a natural spline with 3 degrees of freedom. We accounted for the potential nonlinear effect of daily mean ambient temperature using a cubic B-spline with 3 internal knots, placement of which was evaluated based on model AIC and guided by recent literature [36]. We compared cubic B-splines with 3 internal knots placed at the 10th, 50th and 90th vs. 10th, 75th, and 90th percentiles of city-specific temperature distributions, and found that the AIC was lowest for the latter. Infant sex, maternal age (19 years or less, 20–39, 30–39, 40+ years), maternal marital status (single, married, separated, divorced, widowed), maternal country of birth (Canada, elsewhere), tertile of neighbourhood percent unemployed, low income, visible minority, and with post-secondary education, indicator variables for year and season of birth and a natural spline function of day of year with 3 degrees of freedom were included as covariates in each city specific model. Subgroup analyses were conducted by infant sex, gestational age category (20–27, 28–31, 32–33, 34–36 weeks), tertile of neighbourhood percent low income and season. In the second stage, we pooled the estimated city-specific hazard ratios using a random effects model. Associations with *p*-values< 0.05 were considered statistically significant. Analyses were performed with R version 3.4, using dlnm package, version 2.3.2 and metafor, version 2.0.

## Results

During the study period there were 1,248,240 singleton births in the 24 cities. Frequency and prevalence of preterm and term birth by maternal and infant characteristics, city, season and year are shown in Table 1. Maternal age 19 years and under or 40 years and over, and maternal marital status of single, divorced and separated were associated with a higher prevalence of preterm birth. St. John’s, Winnipeg, Calgary and Edmonton had the highest prevalence of preterm birth. There was no apparent trend by year or season. After exclusion of births with missing covariate data, 1,001,700 births were included in the analysis including 63,400 preterm births, resulting in an overall prevalence of preterm birth of 6.34%. (Note that in accordance with Statistics Canada disclosure rules, all frequencies were randomly rounded to base five. Statistical analyses employed unrounded data.)

**Table 1:** Preterm and term births by maternal and infant characteristics, city, season and year

Variable	Number of Births^a^			Percent Preterm
	Preterm	Term	Total	
Maternal age (years)				
19 or less	3515	38,690	42,200	8.33
20–29	33,795	501,050	534,845	6.32
30–39	39,760	587,780	627,540	6.34
40+	3800	39,765	43,565	8.72
Missing	10	75	90	11.11
Maternal marital status				
Single	18,080	223,090	241,170	7.50
Married	51,410	809,340	860,755	5.97
Widowed	70	965	1035	6.76
Divorced	1050	12,175	13,225	7.94
Separated	330	3345	3675	8.98
Missing	9935	118,450	128,385	7.74
Parity				
0	41,025	546,435	587,460	6.98
1	23,890	405,325	429,215	5.57
2	15,775	213,305	229,080	6.89
Missing	190	2300	2490	7.63
Maternal country of birth				
Other	33,855	500,350	534,205	6.34
Canada	45,085	642,225	687,310	6.56
Missing	1935	24,790	26,725	7.24
Infant sex				
Male	44,380	596,410	640,790	6.93
Female	36,495	570,950	607,445	6.01
Missing	5	5	10	50.00
City				
St. John’s	725	8570	9295	7.80
Saint John	455	6760	7215	6.31
Fredericton	335	4620	4955	6.76
Quebec	2630	37,975	40,605	6.48
Trois Rivieres	625	9690	10,315	6.06
Montreal	11,350	170,830	182,180	6.23
Ottawa	5110	76,605	81,715	6.25
Oshawa	855	12,845	13,700	6.24
Toronto	17,395	265,895	283,290	6.14
Mississauga	4340	72,490	76,830	5.65
Brampton	3695	52,325	56,020	6.60
Hamilton	3205	44,960	48,165	6.65
St. Catharines	690	11,390	12,085	5.71
Kitchener	1370	21,600	22,970	5.96
Windsor	1445	21,885	23,330	6.19
Winnipeg	4965	64,845	69,810	7.11
Calgary	8980	111,425	120,405	7.46
Edmonton	6630	78,655	85,285	7.77
Richmond	835	14,630	15,470	5.40
Vancouver	3475	51,610	55,085	6.31
Victoria	375	5805	6180	6.07
Nanaimo	425	6385	6810	6.24
Kamloops	440	6940	7385	5.96
Kelowna	525	8620	9150	5.74
Birth year				
1999	7660	113,210	120,875	6.34
2000	7980	113,205	121,185	6.58
2001	7810	115,315	123,130	6.34
2002	7750	113,460	121,210	6.39
2003	6880	100,615	107,495	6.40
2004	8115	115,690	123,805	6.55
2005	8365	118,735	127,100	6.58
2006	8505	122,110	130,615	6.51
2007	8720	125,625	134,340	6.49
2008	9090	129,395	138,485	6.56
Season				
Spring (Apr-Jun)	20,725	300,785	321,510	6.45
Summer (Jul-Sep)	20,195	305,135	325,330	6.21
Autumn (Oct-Dec)	19,850	280,030	299,880	6.62
Winter (Jan-Mar)	20,110	281,415	301,525	6.67
Gestation (weeks)				
20–27	4590	0	4590	.
28–31	6580	0	6580	.
32–33	8735	0	8735	.
34–36	60,970	0	60,970	.
37+	0	1,167,365	1,167,365	.
Total	80,875	1,167,365	1,248,240	6.48
Total (excluding missing covariates)	63,400	938,300	1,001,700	6.34

^a^In accordance with Statistics Canada disclosure rules, case counts of less than five were suppressed, and all frequencies were randomly rounded to base five. As a result, there may be discrepancies between column totals and totals by infant/maternal characteristic. Statistical analyses employed unrounded data

The combined population of the 24 cities was 11,522,776 in 2006. A descriptive summary of air pollution and temperature data is shown in Table 2. Mean PM_2.5_ concentrations were highest in Montreal, Hamilton and Windsor in relation to traffic and industrial activity, while maxima were highest in Kamloops and Kelowna due to wildfire smoke. Mean NO_2_ concentrations, an indicator of traffic pollution, were greatest in Vancouver, Calgary and Toronto, while mean and maximum ozone concentrations were generally highest in the southwestern Ontario cities of Brampton, Hamilton, St. Catharines and Kitchener, consistent with the most common location of summer regional smog events. Mean SO_2_ concentrations were highest in Saint John, Montreal, Hamilton and Windsor, reflecting local industrial activity, and CO concentrations were uniformly low. Mean temperatures were generally mildest and exhibited the narrowest ranges in the coastal British Columbia cities of Richmond, Vancouver, Victoria and Nanaimo.

**Table 2:** Summary of population, air pollution and temperature data by city

City	2006 population	PM_2.5_ (μg/m^3^)^a^				NO_2_ (ppb)^a^				O_3_ (ppb)^a^				SO_2_ (ppb)^a^				CO (ppm)^a^				T (°C)^a^			
		*N*	mean	min	max	*n*	mean	min	max	*n*	mean	min	max	*n*	mean	min	max	*n*	mean	min	max	*n*	mean	min	max
St John’s	100,646	3470	4.6	0.0	49.0	3040	7.8	0.1	57.8	3620	25.0	2.1	57.5	3105	2.5	0.0	19.7	3605	0.4	0.0	1.9	3655	6.2	−15.3	24.6
Saint John	68,043	3350	6.8	0.0	109.1	3575	7.4	0.0	59.4	3655	25.0	3.0	72.7	3645	4.4	0.0	63.9	3510	0.5	0.0	3.3	3655	5.6	−23.4	23.6
Fredericton	50,535	3365	5.9	0.0	42.0	3190	4.9	0.0	34.8	3460	24.6	2.4	56.1	0	.	.	.	3470	0.3	0.0	1.9	3645	6.7	−23.8	28.2
Quebec	491,142	3360	10.1	0.0	111.9	3465	12.8	0.4	56.9	3655	21.1	1.0	57.2	3440	2.1	0.0	26.4	3480	0.4	0.0	2.6	3655	5.3	−28.2	28.5
Trois Rivieres	126,323	3540	9.7	0.0	66.3	0	.	.	.	3460	21.2	0.7	58.5	3630	3.0	0.0	36.5	0	.	.	.	3655	6.0	−27.3	28.5
Montreal	1,620,693	3650	11.4	0.0	83.4	3655	18.3	3.8	57.9	3655	19.1	0.9	68.0	3640	4.4	0.0	28.4	3655	0.4	0.0	2.1	3655	7.3	−26.4	29.2
Ottawa	812,129	3435	8.5	0.0	70.0	3575	14.7	0.0	57.0	3635	22.8	0.8	66.2	3640	2.4	0.0	17.0	3640	0.5	0.0	2.0	3655	7.0	−26.7	30.4
Oshawa	141,590	3610	9.8	0.0	63.5	3180	15.3	0.3	52.9	3610	25.1	2.5	66.5	610	3.3	0.0	16.4	365	0.7	0.0	2.1	3655	8.6	−21.0	29.0
Toronto	2,503,281	3655	10.6	0.0	64.9	3650	21.9	4.8	62.3	3650	21.7	2.4	65.6	3650	3.2	0.0	19.6	3645	0.6	0.0	2.5	3655	9.2	−19.6	30.3
Mississauga	668,549	3580	10.1	0.0	67.8	1855	19.8	3.2	58.9	3590	22.6	0.7	75.6	2670	3.4	0.0	42.0	2245	0.7	0.0	2.7	3655	8.9	−20.3	31.5
Brampton	433,806	3070	9.9	0.0	69.2	3035	16.2	1.8	58.1	3085	25.6	0.5	78.5	2365	2.1	0.0	17.4	1590	0.8	0.0	3.0	3655	8.3	−20.5	30.8
Hamilton	504,559	3595	11.5	0.0	64.2	3575	17.7	1.0	62.6	3595	23.8	0.0	84.6	3580	5.1	0.0	35.6	3335	0.5	0.0	2.0	3655	8.2	−19.6	29.6
St. Catharines	131,989	3555	10.2	0.0	63.5	2515	13.9	2.1	77.6	3555	24.3	0.0	81.0	1705	2.9	0.0	19.2	1305	0.3	0.0	1.4	3655	9.7	−15.0	30.0
Kitchener	204,668	3360	9.8	0.0	67.8	3305	12.2	0.8	53.2	3580	26.4	0.8	82.7	2080	2.9	0.0	16.8	1750	0.4	0.0	1.6	3650	7.3	−22.0	30.0
Windsor	216,473	3475	11.9	0.5	68.0	3490	18.4	2.9	55.7	3590	22.7	0.5	76.9	3590	5.9	0.0	30.5	3365	0.4	0.0	2.4	3655	10.6	−16.8	31.5
Winnipeg	633,451	3650	7.1	0.0	36.8	3650	11.8	1.1	39.3	3655	19.9	1.2	51.0	0	.	.	.	3645	0.4	0.0	1.6	3640	4.6	−33.0	30.0
Calgary	988,193	3635	9.1	0.7	90.7	3655	21.3	4.9	63.7	3655	18.9	0.6	51.1	3625	2.1	0.0	14.2	3655	0.5	0.2	2.4	3655	4.9	−31.6	24.7
Edmonton	730,372	3655	9.3	0.0	74.0	3650	20.8	3.2	62.9	3653	18.4	0.5	48.6	0	.	.	.	3655	0.5	0.1	2.7	3655	4.0	−34.7	26.5
Richmond	174,461	3570	6.8	0.0	44.0	3655	16.7	3.6	40.5	3655	15.2	0.3	44.9	3580	1.0	0.0	4.5	3655	0.5	0.1	2.3	3655	10.6	−8.6	24.2
Vancouver	578,041	3565	6.9	1.0	36.3	3655	22.5	4.7	43.2	3655	10.0	0.0	37.5	3655	3.3	0.0	18.1	3655	0.6	0.1	2.4	3655	11.1	−7.0	25.0
Victoria	78,057	3160	7.3	0.0	37.9	2770	11.1	0.5	30.9	3215	17.9	0.2	46.4	2945	1.3	0.0	16.5	3055	0.5	0.0	2.1	3650	10.2	−7.2	26.7
Nanaimo	78,692	3620	5.5	0.0	24.5	865	8.4	1.1	20.1	3600	19.2	0.1	45.0	1055	1.0	0.0	3.8	0	.	.	.	3655	10.4	−11.0	27.0
Kamloops	80,376	3595	6.9	0.0	140.0	3545	10.6	0.1	38.2	3600	20.9	0.0	52.4	3645	0.5	0.0	5.4	2895	0.2	0.0	1.4	3655	9.4	−24.2	29.8
Kelowna	106,707	3590	6.9	0.0	186.0	3345	9.0	0.2	35.3	3615	21.0	0.0	51.1	2915	0.2	0.0	2.8	2915	0.3	0.0	1.7	3650	8.9	−24.2	28.5

^a^24 h average values

**Fig. 1:**
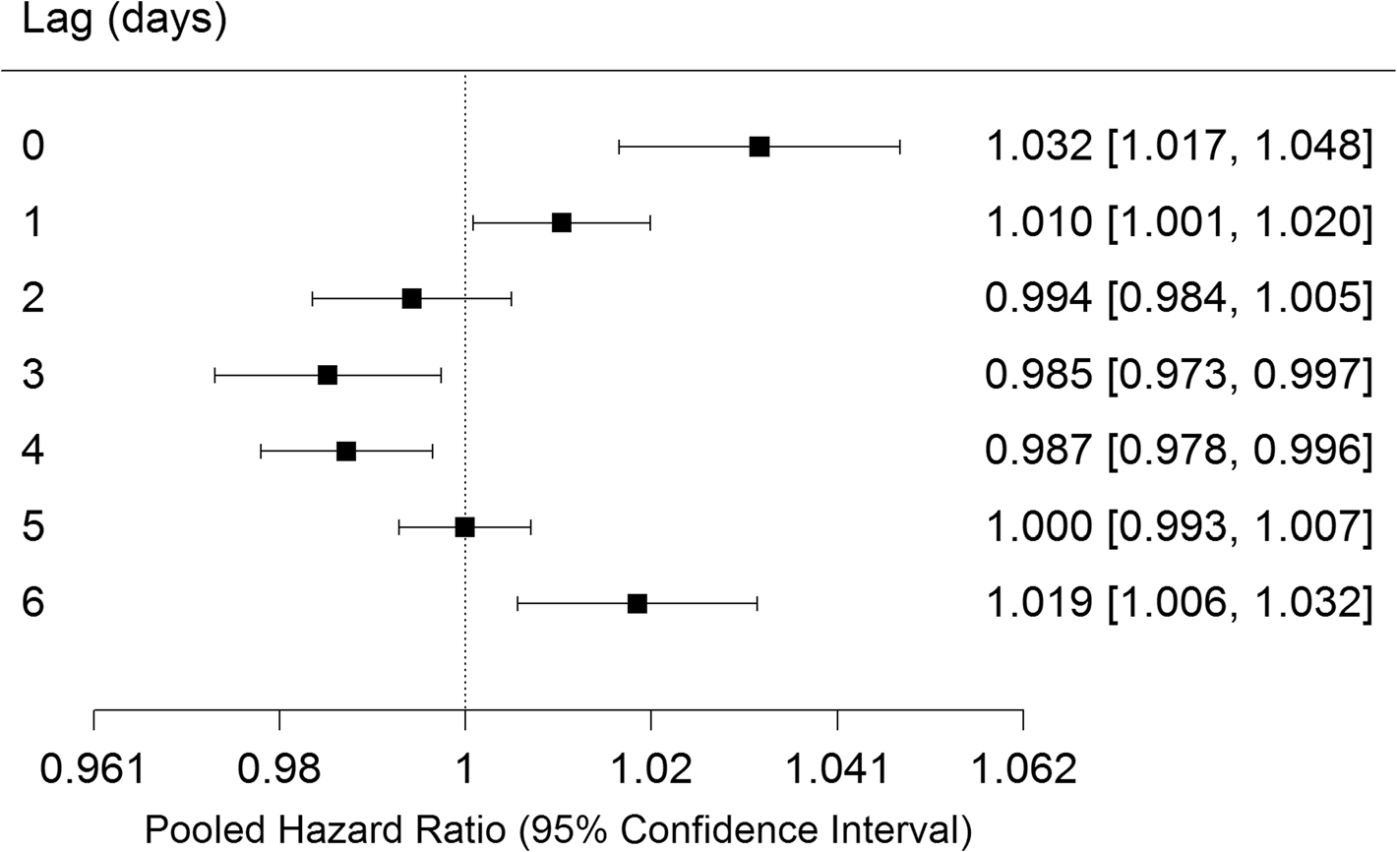
Pooled hazard ratios, 95% confidence intervals by single day lag, distributed lag models. Expressed per 13.3 ppb O_3_ (interquartile range)

Pooled estimates of associations with O_3_ by lag day from distributed lag models are shown in Fig. 1. The lag 0, 1 and 6 day Hazard Ratios (HR) were positive and significant, while lags 3 and 4 days were negative and significant. I^2^, Cochrane’s Q and p-values of Q are shown in Table 3. There was significant heterogeneity between cities only for lag 2 days. The cumulative lag HRs for 0–1, 0–2 and 0–3 days were significant. Results for individual cities at lag 0 days are shown in Additional file 1: Figure S1. Significant positive associations were observed in Toronto (HR 1.038 95% CI 1.009, 1.067), Mississauga (HR 1.057 95% CI 1.005, 1.111), Quebec City (HR 1.075 95% CI 1.004, 1.151), Edmonton (HR 1.096 95% CI 1.040, 1.156) and Windsor (HR 1.131 95% CI 1.035, 1.236) (all are expressed per 13.3 ppb O_3_). As a sensitivity analysis, we specified O_3_ as a natural spline function with 3 degrees of freedom in four cities of varying sizes and climates (Vancouver, Edmonton, Winnipeg and Toronto) and found that in all cases models employing a linear O_3_ term had a lower AIC, indicating better fit.

**Table 3:** Pooled hazard ratios, 95% confidence intervals and heterogeneity measures from distributed lag models

Lag	Hazard Ratio^a^	95% confidence interval^a^		*p*	I^2^	Q	p(Q)
0	1.032	1.017	1.048	<.0001	12.83%	26.3845	0.2831
1	1.010	1.001	1.020	0.0327	26.81%	31.4232	0.1127
2	0.994	0.984	1.005	0.2897	38.81%	37.5859	0.0282
3	0.985	0.973	0.997	0.0171	33.68%	34.6821	0.0559
4	0.987	0.978	0.996	0.0066	23.55%	30.0833	0.147
5	1.000	0.993	1.007	0.9832	0.00%	18.9691	0.703
6	1.019	1.006	1.032	0.005	0.00%	22.7601	0.4749
0–1	1.032	1.016	1.049	<.0001	16.81%	27.649	0.2293
0–2	1.042	1.016	1.068	0.0012	23.37%	30.0158	0.1489
0–3	1.036	1.005	1.067	0.0209	29.10%	32.4378	0.0914
0–4	1.022	0.987	1.057	0.227	33.94%	34.8155	0.0543
0–5	1.009	0.971	1.049	0.6396	34.61%	35.1717	0.05
0–6	1.006	0.971	1.042	0.7587	20.63%	28.9787	0.181

^a^Per 13.3 ppb O_3_ (interquartile range)

**Fig. 2:**
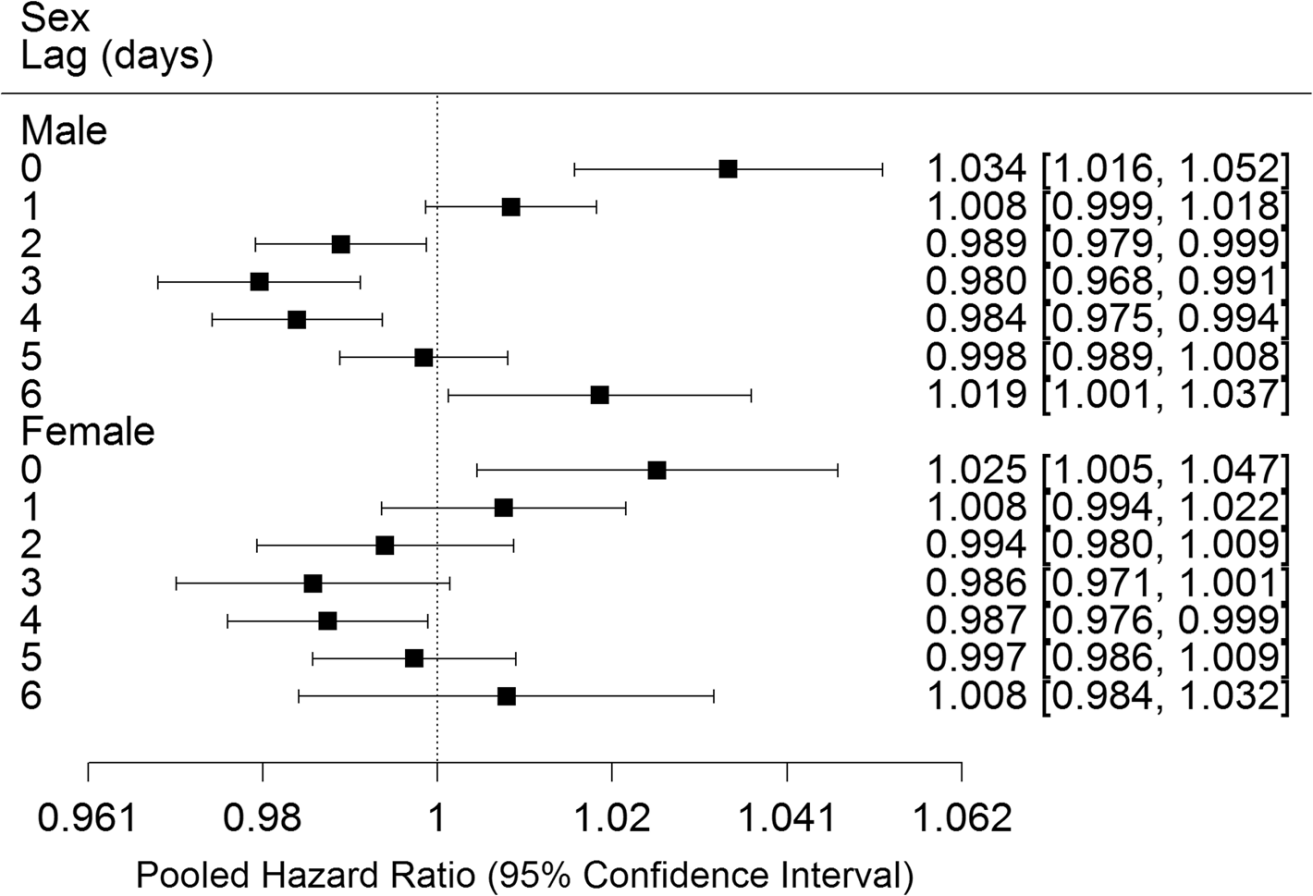
Pooled hazard ratios, 95% confidence intervals by infant sex, single day lag, distributed lag models. Expressed per 13.3 ppb O_3_ (interquartile range)

**Fig. 3:**
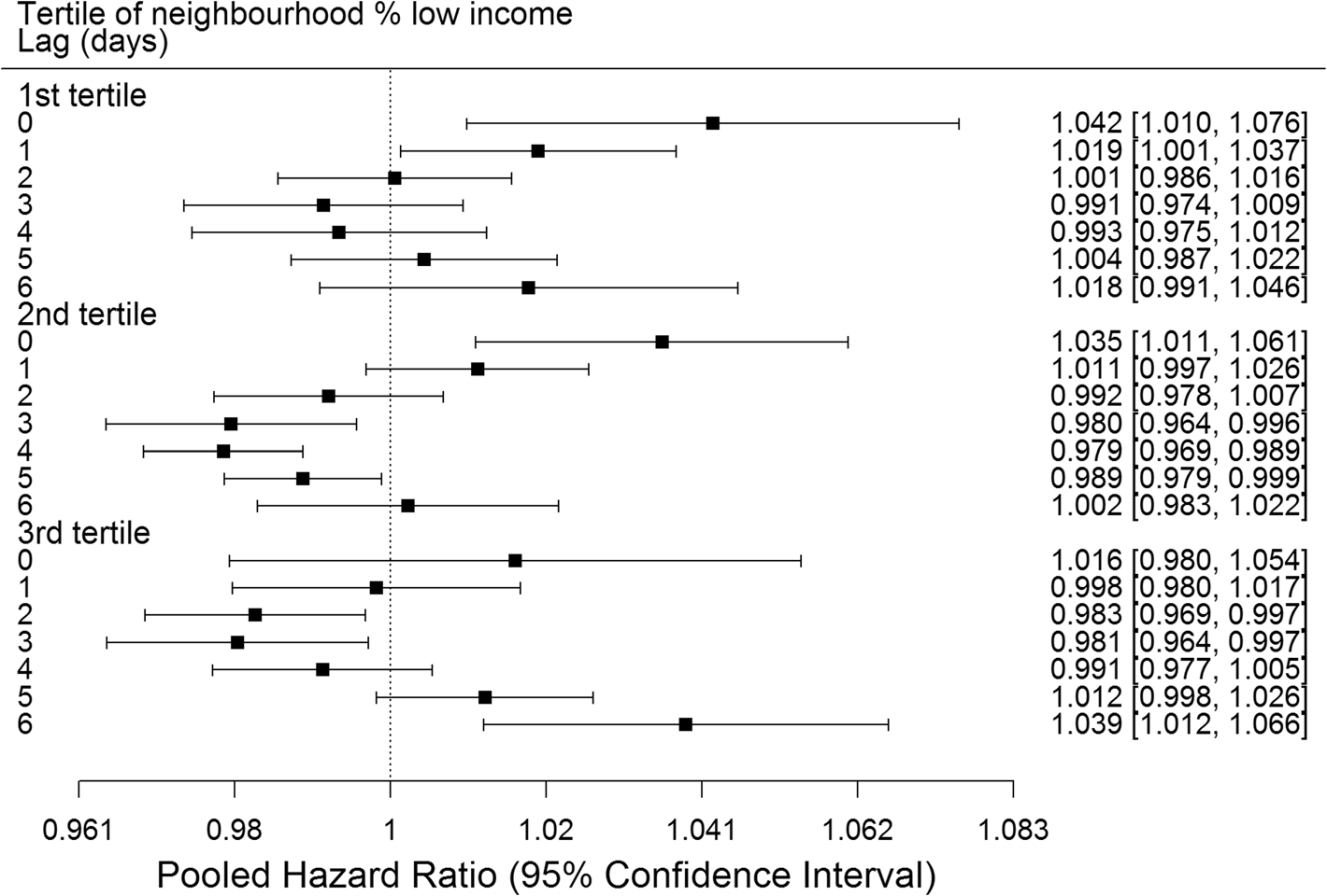
Pooled hazard ratios, 95% confidence intervals by tertile neighbourhood percent low income, single day lag, distributed lag models. Expressed per 13.3 ppb O_3_ (interquartile range)

**Fig. 4:**
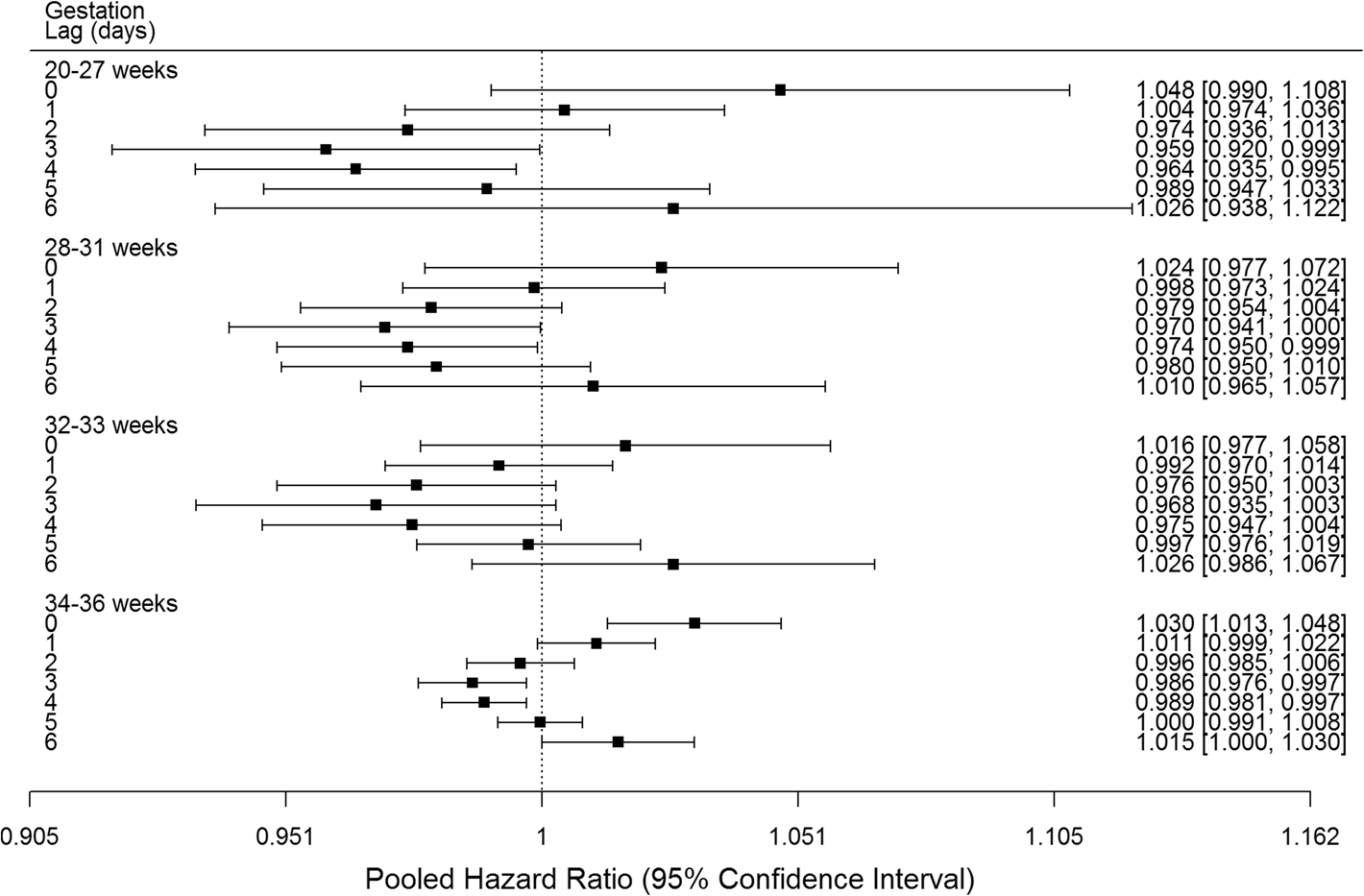
Pooled hazard ratios, 95% confidence intervals by gestational age, single day lag, distributed lag models. Expressed per 13.3 ppb O_3_ (interquartile range)

**Fig. 5:**
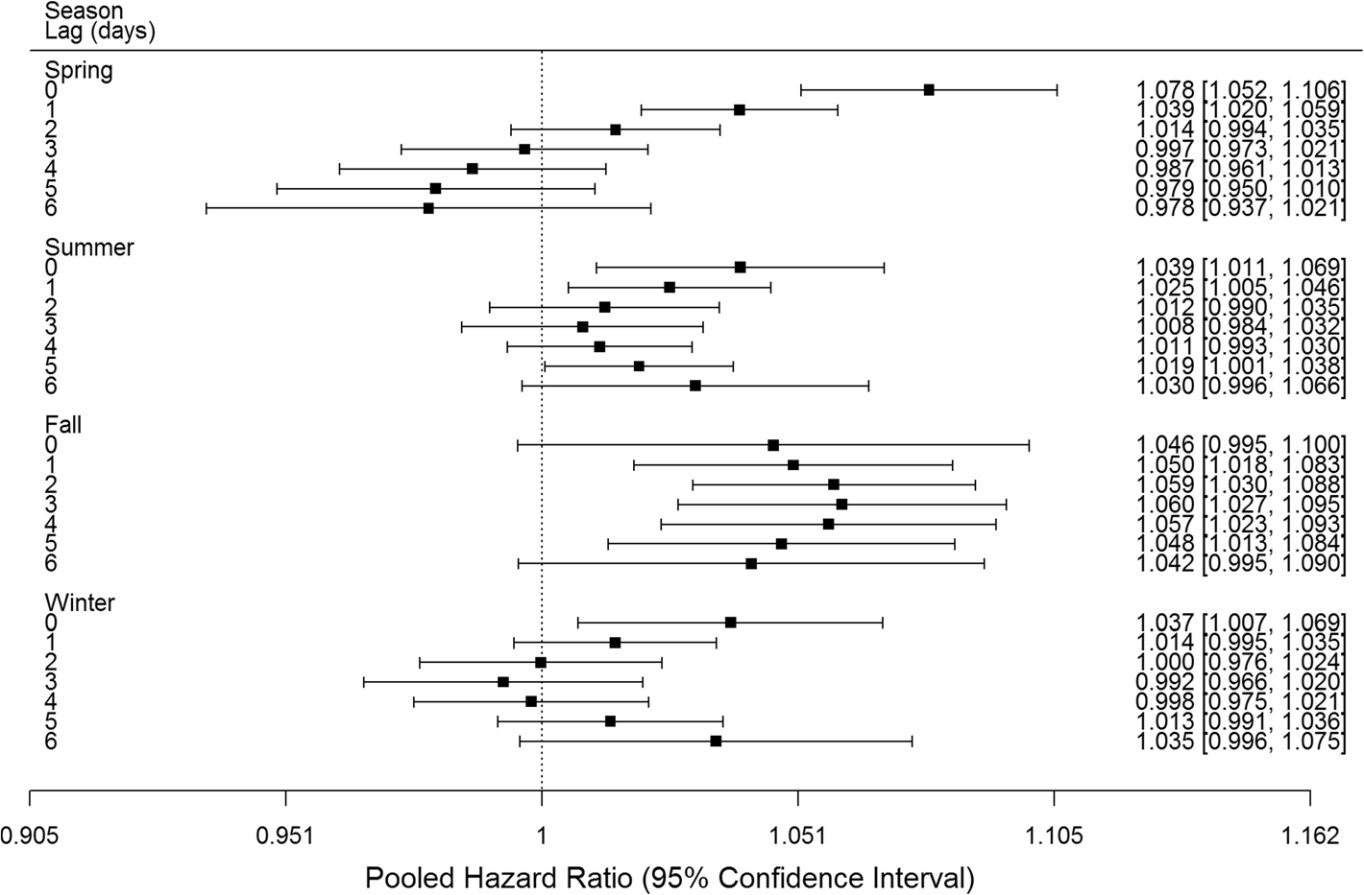
Pooled hazard ratios, 95% confidence intervals by season, single day lag, distributed lag models. Expressed per 13.3 ppb O_3_ (interquartile range)

Analyses by subgroups revealed similar results by lag day for male and female infants (Fig. 2). Significant positive associations were observed of O_3_ with preterm birth at lags 0 and 1 days in the 1st tertile, lag 0 days in the 2nd tertile and lag 6 days in the 3rd tertile of neighbourhood percent low income (Fig. 3). Significant positive associations at lag 0 days were observed for births at 34–36 weeks, while no significant positive associations were observed for births at 20–27, 28–31 or 32–33 weeks (Fig. 4). Significant positive associations were observed at multiple lags in spring, summer and fall, and only at lag 0 in winter (Fig. 5).

Associations with other pollutants were mixed (Additional file 1: Figures S2-S5). CO and NO_2_ exhibited significant negative associations with preterm birth at lag 0, 1, 5 and 6 days and 0, 1 and 6 days respectively, PM_2.5_ exhibited no significant associations, and SO_2_ exhibited significant negative associations at lag 0 and 1 day.

## Discussion

We observed associations between daily O_3_ in the week prior to delivery and preterm birth in an analysis of approximately 1 million births in 24 Canadian cities between 1999 and 2008. Our findings for PM_2.5_ and NO_2_ were similar to our earlier analysis where we found null or negative associations of preterm birth with PM_2.5_ or NO_2_ averaged over gestational month, trimester or the entire pregnancy [12, 13]. Associations were similar for male and female infants but differed by gestational age and season. Our observation of significant associations only at longer gestational ages may result from greater statistical power afforded by the larger number of pregnancies in these categories of gestational age. Greater time spent outdoors and/or increased indoor penetration of outdoor pollutants in spring, summer and autumn could explain our observation of significant positive associations over multiple lags during these seasons, but only for a single lag in winter. Associations with other pollutants were inconsistent.

Our analysis is one of a limited number which have examined these short term associations employing Cox proportional hazards models to account for the different exposure durations of preterm vs. term births (in contrast to studies based on exposure during the entire pregnancy or third trimester). O_3_ exposure in particular has received relatively little attention in previous studies. In their analysis of 13 birth cohorts comprising 71,493 births from the European Study of Cohorts for Air Pollution Effects (ESCAPE), Giorgis-Allemand et al. found no association of preterm birth with NO_2_, nitrogen oxides (NO_x_), PM_2.5_ and PM_10_ exposures over durations ranging from one week to the entire pregnancy [22]. In an analysis of 78,633 births in Rome and 27,255 in Barcelona, Schifano et al. found that PM_10_ and NO_2_ in the week prior to delivery were positively associated with preterm birth in Barcelona and negatively associated with preterm birth in Rome, while ozone was positively associated with preterm birth in both cities [23]. The hazard ratios for O_3_ were comparable in magnitude to what we observed: 1.010 (95% CI 1.001, 1.020) per 9.2 ppb in Barcelona and 1.025 (95% CI 1.009, 1.042) per 15.3 ppb in Rome [23]. In contrast to our findings, they observed larger associations at shorter pregnancy durations [23]. An earlier study by the same authors examining preterm birth in Rome using time-series methods found that PM_10_, O_3_ and NO_2_ lagged 0–2 days were not associated with preterm birth in the warm or cold season; only PM_10_ lagged 12–22 days in the warm season was significantly associated with preterm birth [19]. In a study of nearly 500,000 births in Guangzhou, significant associations were observed between preterm birth and PM_10_, NO_2_ and O_3_, with the peak magnitude of effect at 25 weeks (HR = 1.048, 95% CI 1.034–1.062 per IQR, 37.0 μg/m^3^), 26 weeks (HR = 1.060, 95% CI 1.028–1.094 per IQR, 15.4 ppb) and 28 weeks (HR = 1.063, 95% CI 1.046–1.081 per IQR, 45.8 ppb) gestation respectively [26]. We recently reported that PM_2.5_ on the day of delivery was associated with preterm birth only among women assigned to the highest quartile of PM_2.5_ glutathione-related oxidative potential based on approximately 200,000 births among 31 cities in the province of Ontario, Canada [25]. Johnson et al. found no association between cumulative third trimester PM_2.5_ or NO_2_ and preterm birth in a discrete time survival analysis of 258,294 births in New York City [24]. Sagiv et al. conducted a time-series analysis of 187,997 births in Pennsylvania and found that preterm birth was associated with PM_10_ 2 days and 5 days before birth (relative risk (RR) = 1.10; 95% CI, 1.00–1.21 per 50 μg/m^3^ and RR = 1.07; 95% CI, 0.98–1.18 per 50 μg/m^3^ respectively) [14] . Associations with O_3_ were not reported. In another time series analysis of 476,489 births in Atlanta, Darrow et al. observed mostly null associations with air pollution (including O_3_), but reported that preterm birth was associated with PM_2.5_ sulfate and PM_2.5_ water-soluble metal concentrations in the week preceding delivery [15]. Rappazzo et al. also reported that PM_2.5_ lagged 0–2 weeks before birth was associated with an increased risk of preterm birth in an analysis of nearly 1.8 million births in Ohio, Pennsylvania, and New Jersey [17]. O_3_ was included as a covariate but associations of preterm birth with O_3_ were not reported. A time series study in Ahvaz, Iran found no association between O_3_ in the two weeks prior to birth and preterm birth, although significant associations with CO, NO_2_ and PM_10_ were observed [20]. Lee et al. reported no associations of O_3_, PM_10_ or meteorological variables with preterm birth in a time series analysis in London examining exposures in the week prior to birth [16]. Arroyo et al. found an association between O_3_ in the twelfth week of gestation and preterm birth in a time-series analysis in Madrid [18]. Finally, employing a novel hierarchical spatiotemporal model, Warren et al. found that O_3_ in weeks 1–5 and PM_2.5_ in weeks 4–22 were associated with increased risk of preterm birth in a study in eastern Texas [37]. In their analysis of air pollution attributable preterm births worldwide, Malley et al. [1] employed an odds ratio of 1.13 (95% CI 1.03, 1.24) per 10 μg/m3 PM_2.5_ based on the meta-analysis by Sun et al. [9], considerably larger than what Sagiv et al. [14] or Schifano et al. [23]observed. It should be noted that there may be substantial differences in other factors that could contribute to preterm birth among these studies, including prenatal care, employment rights of pregnant women, and obstetrical decision-making (e.g. decision to induce labour).

Mechanisms through which exposures in the days prior to delivery might trigger preterm birth are unknown, but may include non-specific processes such as inflammation or oxidative stress, which are known to be associated with both preterm birth [38–41] and air pollution exposure [42]. PM_2.5_ could also trigger preterm birth through cardiovascular mechanisms or effects on endothelial function [42].

Strengths of our study include the large sample size distributed over multiple cities and utilization of Cox models which account for the differing length of exposure in preterm and term births, and distributed lag models which parsimoniously evaluate effects over multiple lags. We also assessed the shape of the exposure response relationship, examined effect modification by infant, maternal and other factors, and considered the effects of other pollutants. Limitations of our study include the lack of data on maternal behavioural risk factors and possible exposure measurement error owing to the limited number of monitors within each city. Since our analysis deals by design with short term temporal variability in air pollution exposure, observed associations are unlikely to be confounded by short-term time invariant risk factors such as smoking. In the only other study employing the same design which included data on maternal smoking, Giorgis-Allemand et al. found that results were not sensitive to inclusion of smoking and other individual characteristics as covariates [22]. Exposure measurement error would be expected to be non-differential, biasing observed associations towards the null [43], and as a secondary pollutant, O_3_ concentrations would be expected to be relatively homogeneous over larger areas compared to pollutants such as NO_2_. Of four other studies with the same design, two with the same limitations with respect to relatively sparse fixed site monitoring data found consistent associations with O_3_ and inconsistent associations with NO_2_ and PM_10_ [23] and consistent associations with PM_10_, NO_2_ and O_3_ [26], while two others employing temporally adjusted land use regression models for NO_2_, NO_x_, PM_2.5_ and PM_10_ [22] and NO_2_ and PM_2.5_ [24], potentially reducing exposure measurement error, found no significant associations with preterm birth [22, 24]. Data were missing for at least one covariate for approximately 20% of births in our study. Marital status was the most common missing covariate, and births for which this was missing had a higher prevalence of preterm birth. This suggests that these births differed from those where all covariate data were available which could have biased our results. Results from individual cities were pooled using a random effects model, which treats estimates from individual cities as originating from separate underlying distributions rather than a single common distribution [44]. Differences between cities may stem from differences in the exposure mix, impact of confounders such as weather, or population characteristics. The random effects model is conservative relative to a fixed effects model in that it assigns greater variance to the overall (pooled) measure of effect by incorporating both within and between study variance [44].

## Conclusions

In this study, one of a small number employing time to event analysis, we observed significant associations between O_3_ in the week prior to delivery and preterm birth, based on an analysis of approximately 1 million births over a ten year period. Given the mixed findings in other studies of this kind, additional studies are needed to determine whether the weight of evidence supports the existence of a causal association between preterm birth and air pollution exposure in the days preceding delivery.

## Additional file

Additional file 1: **Figure S1.** Hazard Ratio by City per 13.3 ppb O_3_ lag 0 days. **Figure S2.** Pooled Hazard Ratio by Lag per 0.36 ppm CO. **Figure S3.** Pooled Hazard Ratio by Lag per 10.3 ppb NO_2._ **Figure S4.** Pooled Hazard Ratio by Lag per 7.4 μg/m^3^ PM_2.5_. **Figure S5.** Pooled Hazard Ratio by Lag per 2.9 ppb SO_2_. (PDF 119 kb)

## Abbreviations

AIC: Akaike Information Criterion
CIs: Confidence intervals
CO: Carbon monoxide
DA: Dissemination Area
ESCAPE: European Study of Cohorts for Air Pollution Effects
HR: Hazard ratio
IQR: Interquartile range
NO_2_: Nitrogen dioxide
O_3_: Ozone
PCCF+: Postal Code Conversion File Plus
PM_2.5_: Particulate matter of median aerodynamic diameter ≤ 2.5 μm
SES: Socioeconomic status
SO_2_: Sulfur dioxide
