# Supplementary material for: Air pollution in the week prior to delivery and preterm birth in 24 Canadian cities: a time to event analysis
Source: Environ Health. 2019 Jan 3;18:1. doi: 10.1186/s12940-018-0440-8 (PMC6318965; doi:10.1186/s12940-018-0440-8)

Figure S1. Hazard Ratio by City per 13.3 ppb O_3 lag 0 days

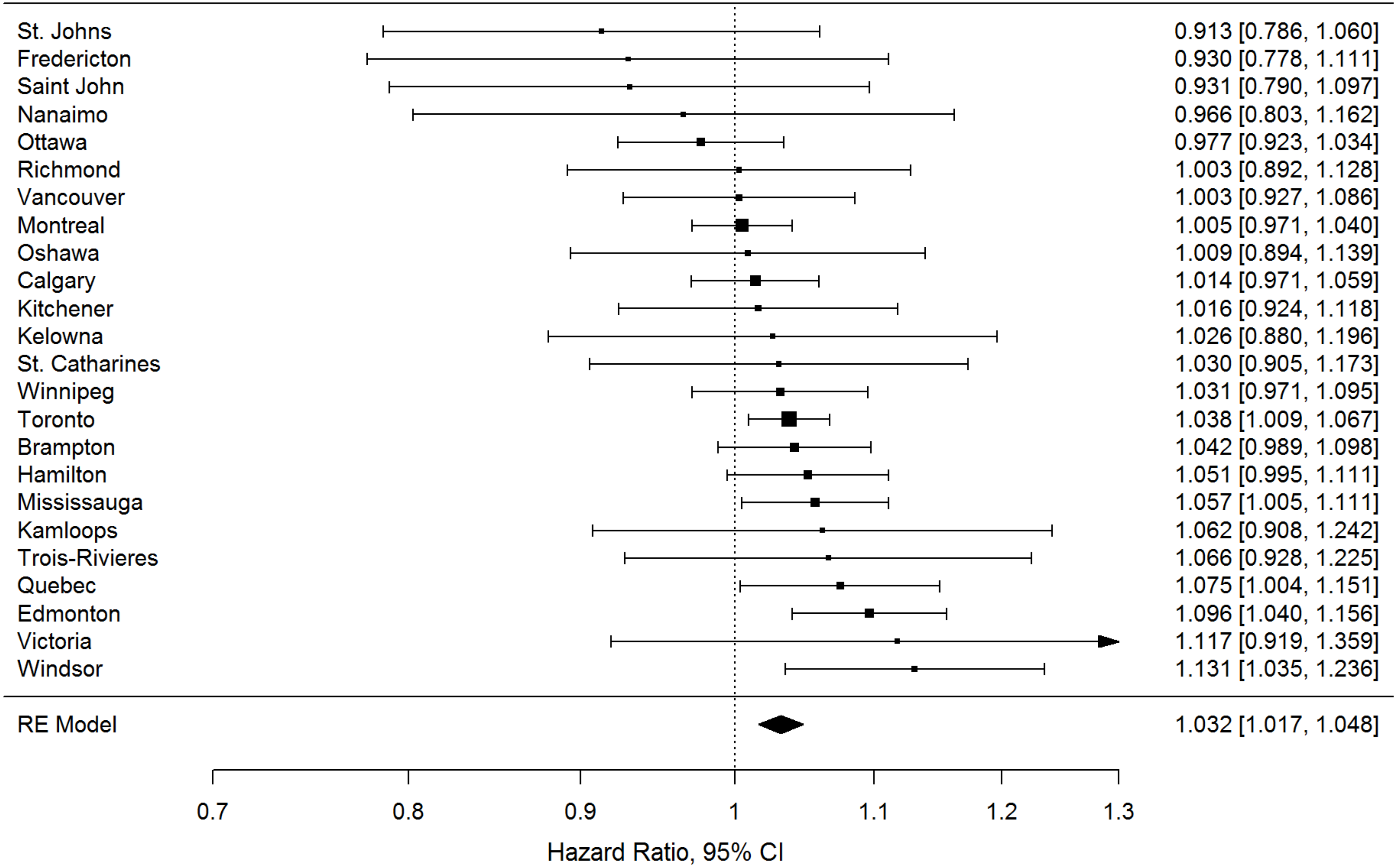

Figure S2. Pooled Hazard Ratio by Lag per 0.36 ppm CO

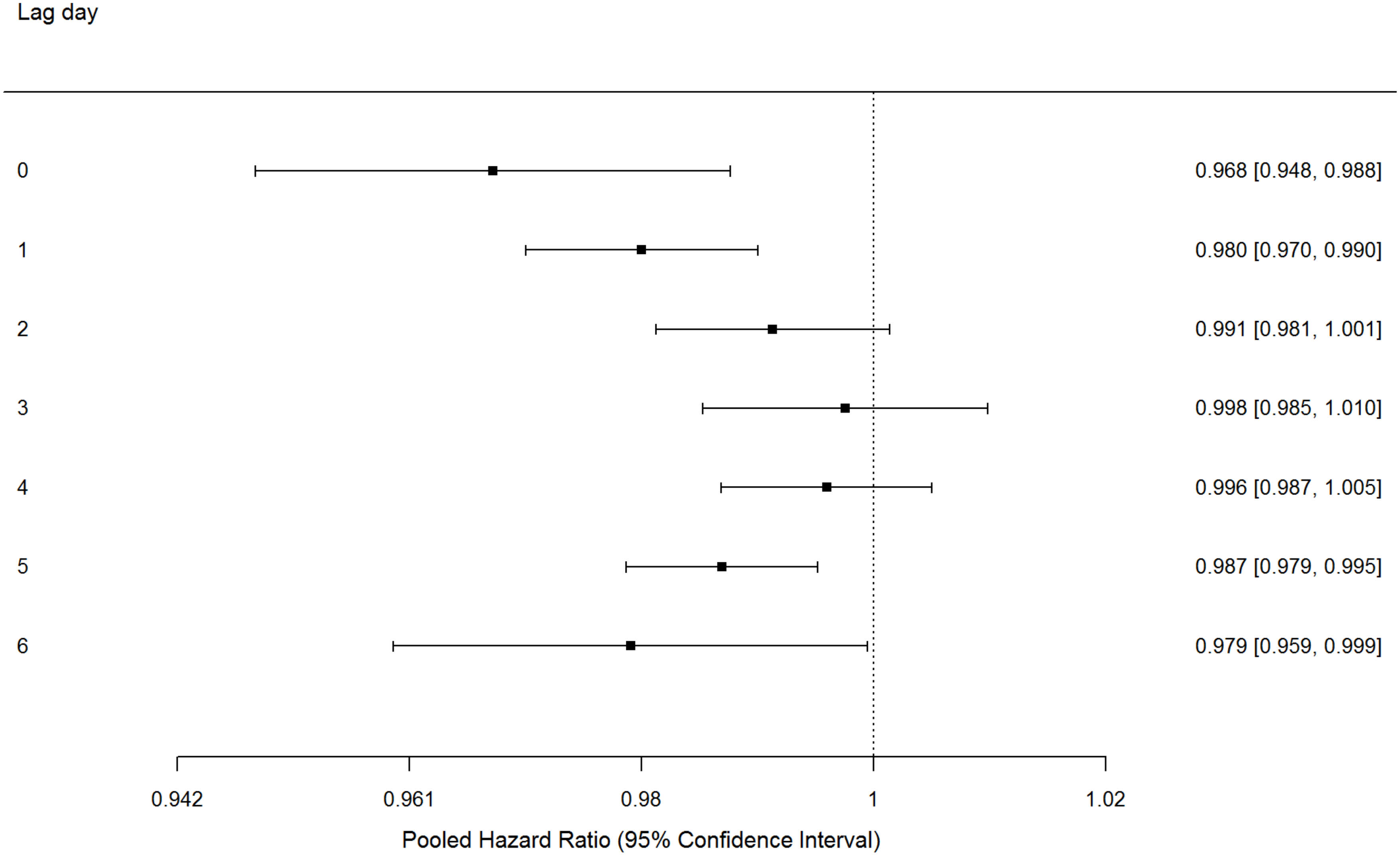

Figure S3. Pooled Hazard Ratio by Lag per 10.3 ppb NO_2

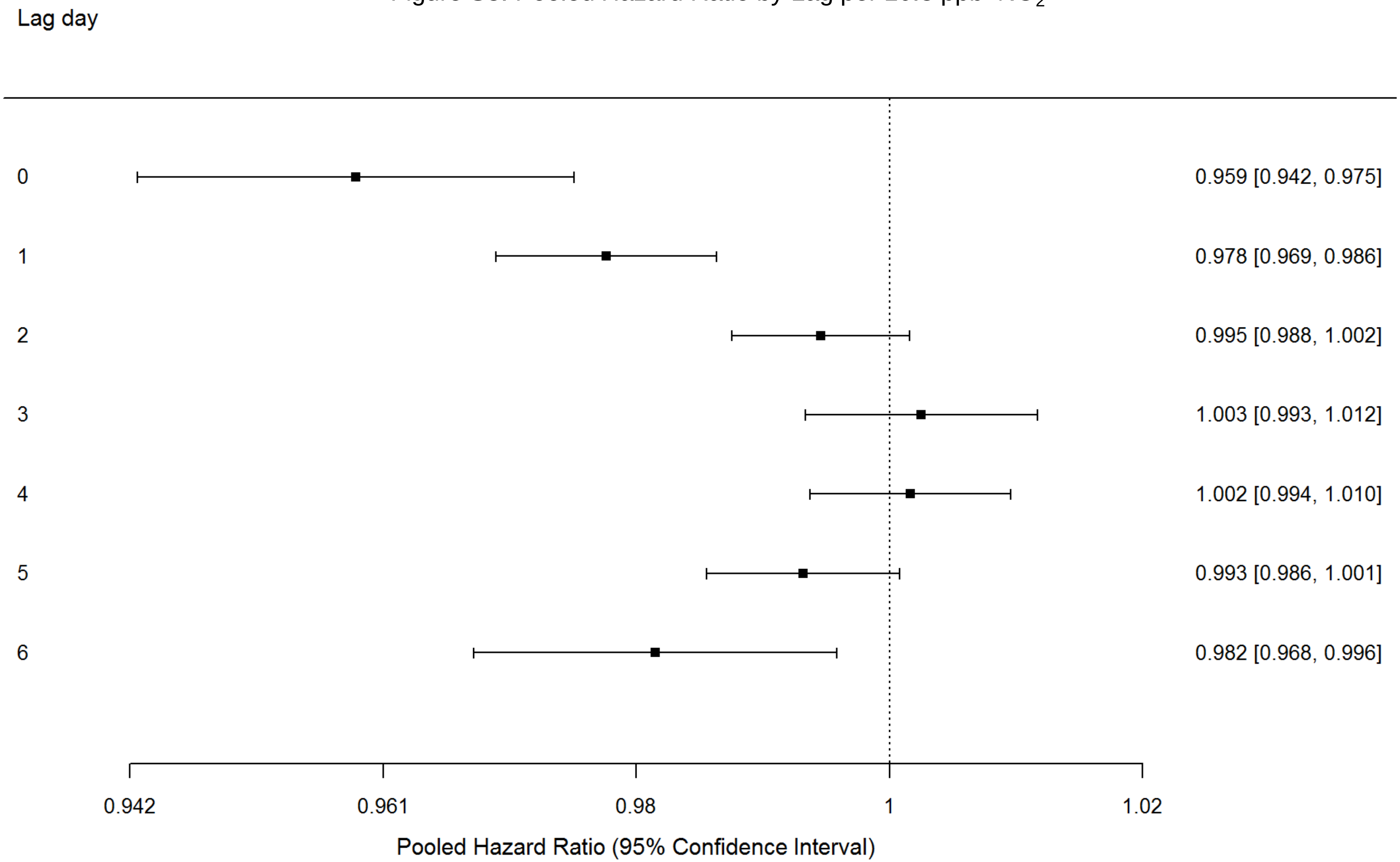

Figure S4. Pooled Hazard Ratio by Lag per 7.4 μg/m^3 $PM_{2.5}$

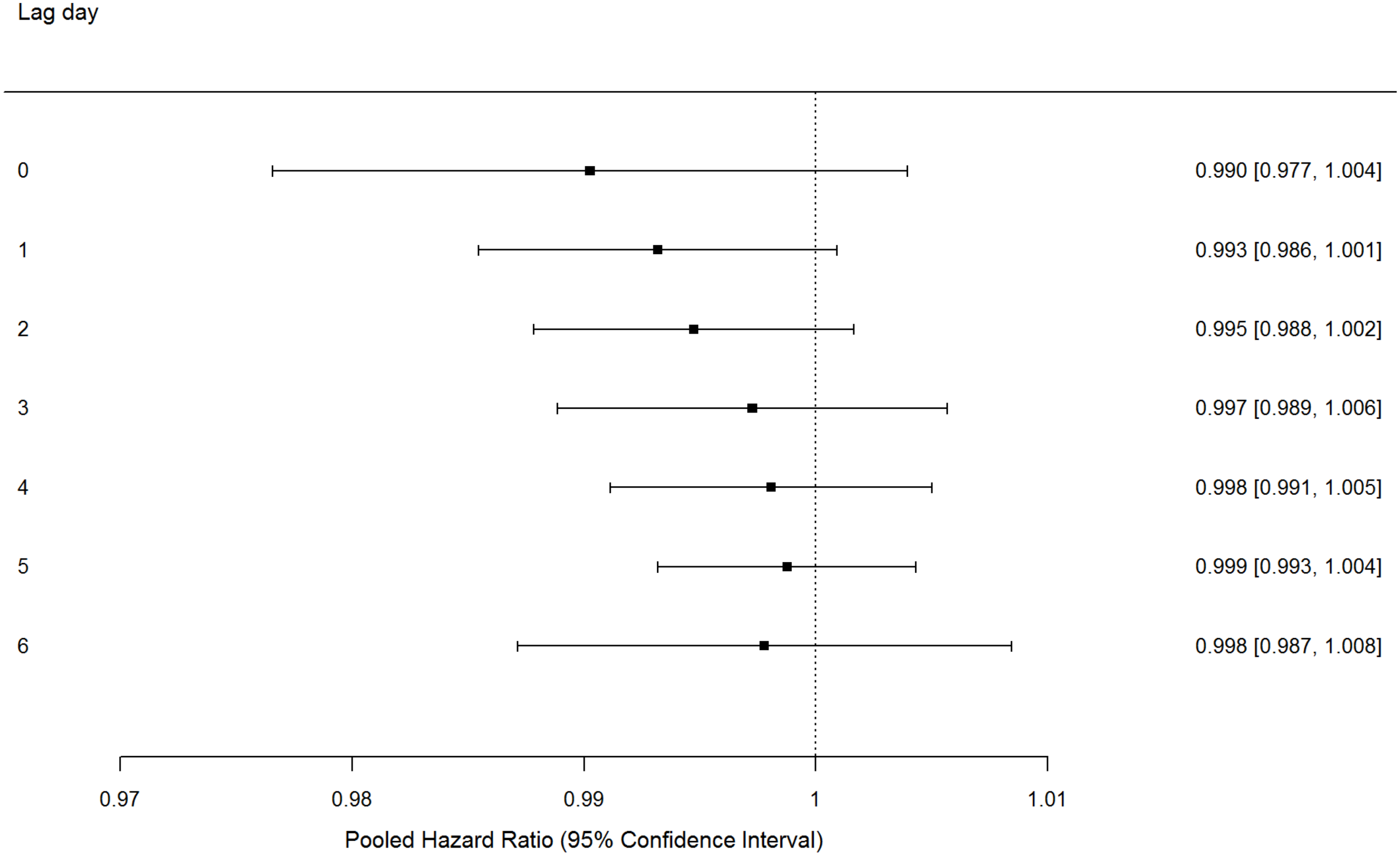

Figure S5. Pooled Hazard Ratio by Lag per 2.9 ppb SO_2

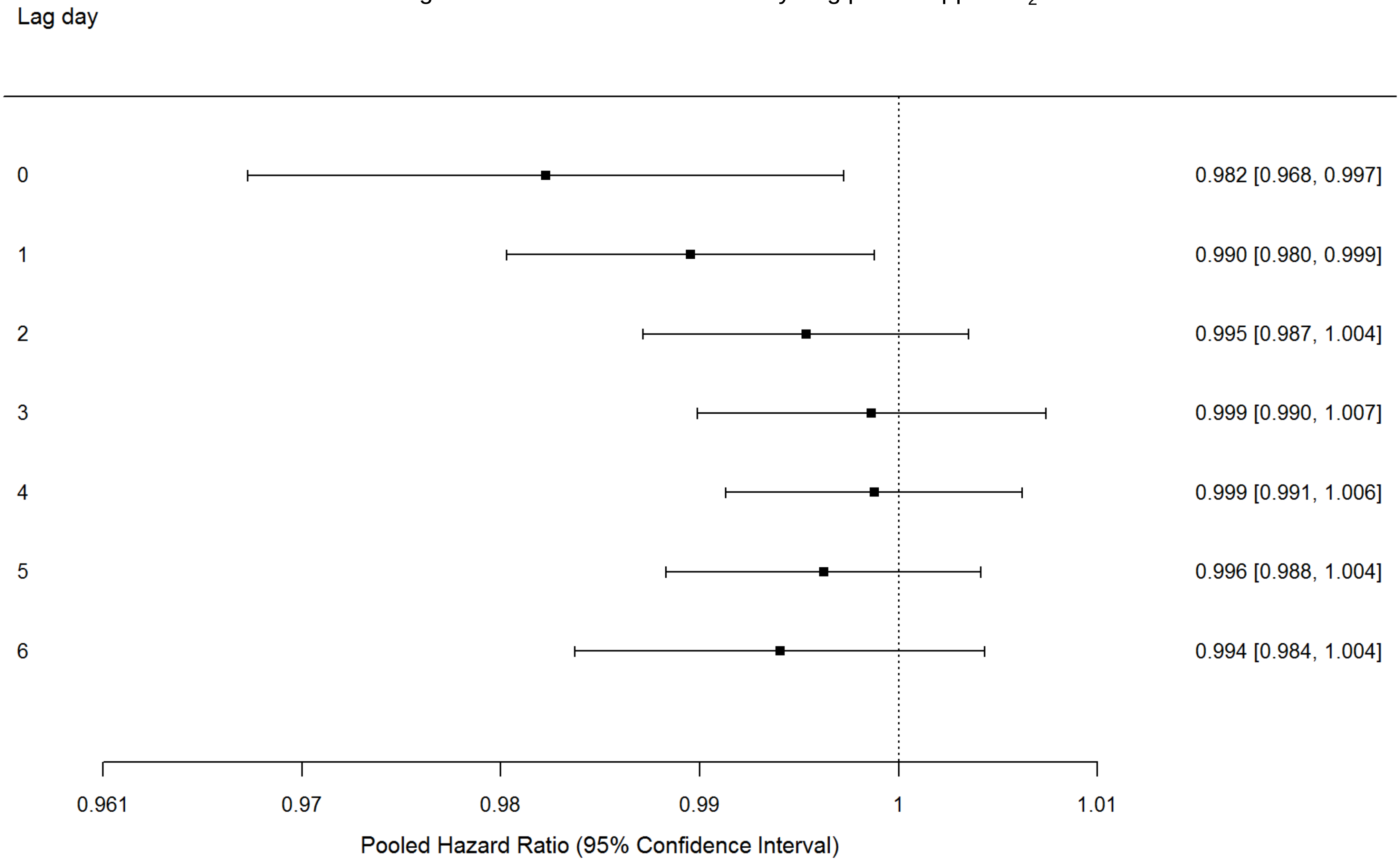

Supplement: Supplementary file 1 — Figure S1. Hazard Ratio by City per 13.3 ppb O3 lag 0 days. Figure S2. Pooled Hazard Ratio by Lag per 0.36 ppm CO. Figure S3. Pooled Hazard Ratio by Lag per 10.3 ppb NO2. Figure S4. Pooled Hazard Ratio by Lag per 7.4 μg/m3 PM2.5. Figure S5. Pooled Hazard Ratio by Lag per 2.9 ppb SO2. (PDF 119 kb) [file 12940_2018_440_MOESM1_ESM.pdf]
